# Analysis of the GGGGCC Repeat Expansions of the *C9orf72* Gene in SCA3/MJD Patients from China

**DOI:** 10.1371/journal.pone.0130336

**Published:** 2015-06-17

**Authors:** Chunrong Wang, Zhao Chen, Fang Yang, Bin Jiao, Huirong Peng, Yuting Shi, Yaqin Wang, Fengzhen Huang, Junling Wang, Lu Shen, Kun Xia, Beisha Tang, Tetsuo Ashizawa, Hong Jiang

**Affiliations:** 1 Department of Neurology, Xiangya Hospital, Central South University, Changsha, Hunan, 410008, P. R. China; 2 Key Laboratory of Hunan Province in Neurodegenerative Disorders, Central South University, Changsha, Hunan, 410008, P. R. China; 3 State Key Laboratory of Medical Genetics of China, Central South University, Changsha, Hunan, 410078, P. R. China; 4 Department of Epidemiology and Biostatistics, School of Public Health, Central South University, Changsha, Hunan, 410078, P. R. China; 5 Department of Neurology, University of Florida, Gainesville, FL, 32610, United States of America; University of Pennsylvania Perelman School of Medicine, UNITED STATES

## Abstract

Neurodegenerative disorders are a heterogeneous group of chronic progressive diseases and have pathological mechanisms in common. A certain causative gene identified for a particular disease may be found to play roles in more than one neurodegenerative disorder. We analyzed the GGGGCC repeat expansions of *C9orf72* gene in patients with SCA3/MJD from mainland China to determine whether the *C9orf72* gene plays a role in the pathogenesis of SCA3/MJD. In our study, there were no pathogenic repeats (>30 repeats) detected in either the patients or controls. SCA3/MJD patients with intermediate/intermediate or short/intermediate genotype (short: <7 repeats; intermediate: 7-30 repeats) of the GGGGCC repeats had an earlier onset compared with those with short/short genotype. The presence of the intermediate allele of the GGGGCC repeats in the patients decreased the age at onset by nearly 3 years. Our study firstly demonstrate that the development of SCA3/MJD may involve some physiological functions of the *C9orf72* gene and provide new evidence to the hypothesis that a specific mutation identified in one of the neurodegenerative disorders may be a modulator in this class of diseases.

## Introductions

Many neurodegenerative disorders, including the following, arise from abnormal protein interactions in the central nervous system: Parkinson’s disease (PD); Alzheimer’s disease (AD); frontotemporal lobar degeneration (FTLD); amyotrophic lateral sclerosis (ALS); and the polyglutamine (polyQ) diseases, including spinocerebellar ataxias (SCA) types 1, 2, 3, 6, 7, and 17, dentatorubral-pallidoluysian atrophy (DRPLA), Huntington’s disease (HD), spinal bulbar muscular atrophy (SBMA), and the recently identified Huntington disease-like 2 (HDL2). These disorders are characterized by adult onset, a chronic progressive course, distinct clinical phenotypes, specific cellular abnormalities, and eventually fatal outcomes. Mutations in the same gene or even the same mutation may perform variable phenotypes among individuals [1]. However, the clinical syndromes from the varied mutations may be strikingly similar to each other [2, 3]. Previous study [4–6] suggested that the CAG repeats mutation in SCA2 or SCA3/Machado-Joseph disease (MJD) may be associated with Parkinsonism. A recent study [7] showed a common molecular mechanism between SCA3/MJD and ALS due to the similar ubiquitination and degradation of ataxia-3 and SOD. A subsequent investigation [8] reported that a patient with a family history of ALS presented symptoms of cerebellar ataxia, which indicates some unclear but subsistent association between SCA and ALS/FTLD. This suggests that there are biological relationships among these genes.

SCA3/MJD, which is the most common dominantly inherited ataxia in China and other countries [9–11], is caused by an unstable CAG trinucleotide repeat expansions in the *ATXN3* gene. SCA3/MJD patients have a clinically heterogeneous presentation with an extreme range of age at onset of 4 years to 70 years [12]. The clinical variability of SCA3/MJD is only partially explained by the CAG repeats in the expanded *ATXN3* alleles, which indicates that the residual variance is likely explained by other unknown factors. GGGGCC repeat expansions, which locates in the first intron of the *C9orf72* gene, was recently identified as a major cause of ALS and FTLD [13]. The pathogenic mechanism of hexanucleotide repeat expansions include interfering with the normal expression of the encoded protein and the loss of protein function through the generation of abnormal toxic RNA foci and the subsequent disruption of normal cellular pathways [14].

Motivated by the observation that different neurodegenerative disorders share some of the same clinical and pathological features, we hypothesized that the genes involved in ALS/FTLD might also play roles in other neurodegenerative disorders. In this study, we investigated a large cohort of SCA3/MJD cases under the hypothesis that the GGGGCC repeat expansions of the *C9orf72* gene may also contribute to the pathogenesis of SCA3/MJD.

## Materials and Methods

### Subjects

A total of 127 SCA3/MJD patients were screened for GGGGCC repeats mutations of the *C9orf72* gene. A total of 314 unrelated, healthy individuals were selected as controls. Both of the groups were enrolled from Xiangya Hospital of Central South University from January 1995 to August 2012. The clinical diagnoses for all SCA3/MJD patients were determined according to the Harding criterion and were confirmed by molecular diagnosis [15]. The age at onset was defined as the age at which the patient, or a close person, noticed the first symptoms (usually gait unbalance). Patients with long-term duration were asked for the age at onset of the mentioned symptoms. To obtain a more accurate age at onset, additional information from previous records and the scores of the International Cooperative Ataxia Rating Scale (ICARS) from at least two experienced neurologists were also taken into account. None of the subjects had cardiac disease, tumors, or other neurological disease. Written informed consent was obtained from all of the individuals. The study was approved by the Expert Committee of Xiangya Hospital of Central South University in China (equivalent to an Institutional Review Board).

### Genetics analysis

Peripheral venous blood samples were drawn from the SCA3/MJD patients and controls and were processed within 4–6 hours to extract the genomic DNA, which was stored at –20°C until further analysis. The genomic DNA was purified from whole blood leukocytes using a DNA extraction kit (QIAGEN, Germany). Recombinant DNA technology, including T-vector cloning, followed by direct DNA sequencing was used to evaluate the size of the CAG repeats in the *ATXN3* gene. We screened the presence of the GGGGCC hexanucleotide expansion of *C9orf72* using a 2-step polymerase chain reaction protocol as previously reported [16]. First, we used a previously reported repeat-primed polymerase chain reaction assay to detect the size of the larger expanded alleles. Then, we performed a classical FAM-fluorescent labeled PCR assay to detect the accurate genotype of the non-pathogenic mutation carriers. The analysis of the PCR fragment length was performed using an ABI 3730xl DNA analyzer (Applied Biosystems, Foster City, CA, USA) and visualized with the GeneMapper software (version 3.2, Applied Biosystems, Foster City, CA, USA). The pathogenic threshold of the GGGGCC repeats was defined as more than 30 [13, 17]. The positive control, which was used to verify the trustworthiness and reliability of our experiment, included a DNA sample from an ALS patient (>30 repeat expansions) recruited from Xiangya Hospital [18].

### Statistics analysis

Univariate linear regression analyses were performed to determine the effect on age at onset of the expanded *ATXN3* allele (linear and quadratic effect) in SCA3/MJD patients. The logarithmically (decimal) transformed age at onset was treated as dependent variables. The differences in the distribution of the GGGGCC repeats size between the SCA3/MJD patients and the controls was tested using the Mann-Whitney U test. The GGGGCC repeats mutations with >30 repeats have also been reported outside of the FTLD and ALS spectrum, such as healthy individuals [19, 20]. Previous study reported that ≥7 units in non-pathogenic carriers are strongly correlated with *C9orf72* expression [21]. Later, a study [16] identified a statistically significant association between the intermediate repeats and PD risk. Thus, in our study, individuals were classified into three genotypes, including S/S, S/I, and I/I (S: short allele<7 units; I: intermediate allele≥7 units) according to an individual’s two repeat alleles. Owing to the small number of individuals with I/I genotypes, we classified subjected into two groups according to the longer allele of the GGGGCC repeats: short allele (the longer allele <7 units, including S/S genotypes) and intermediate allele (the longer allele ≥7 units, including S/I, and I/I genotypes). We employed two analytical approaches for association testing, the larger repeat allele as a continuous variable and two genotyping categorical variables based on the individual’s short or intermediate alleles. Firstly, a partial correlation was used to analyze the role of the GGGGCC repeats size on the age at onset of SCA3/MJD and to control the influence of the expanded CAG in the *ATXN3* allele. Then, a covariance analysis model was used to adjust the effect of expended CAG repeats after interaction analysis. Also, we used multivariate linear regression analyses to test the effect of several potential variables on age at onset, including the CAG repeat size, the *C9orf72* genotypes, and gender. The statistical analyses were performed using the SPSS (version19.0).

## Results

**Fig 1:**
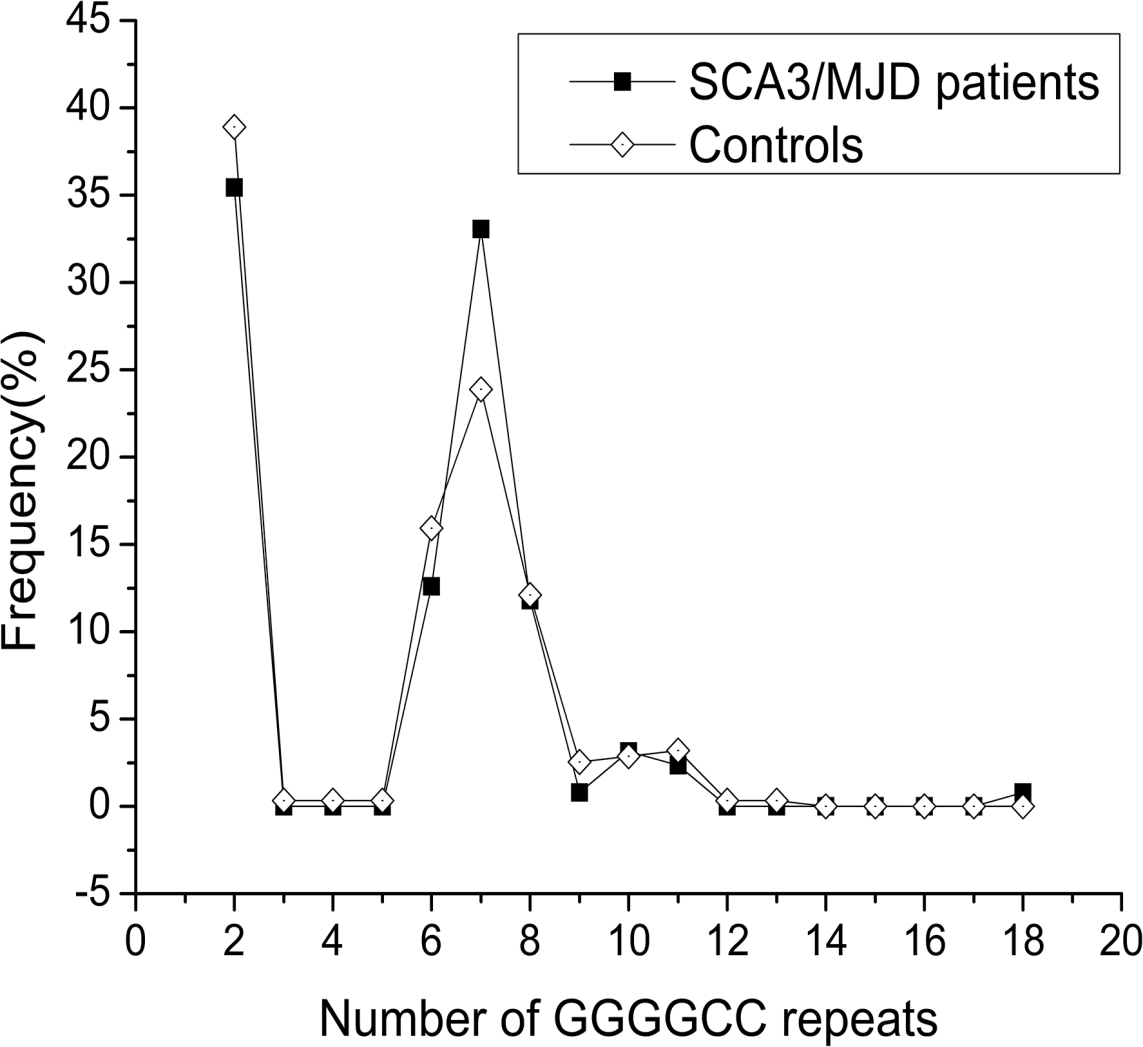
Distribution of the GGGGCC repeats size in SCA3/MJD patients and controls. There was no significant difference in the distribution of the GGGGCC repeats length between SCA3/MJD patients and controls.

**Fig 2:**
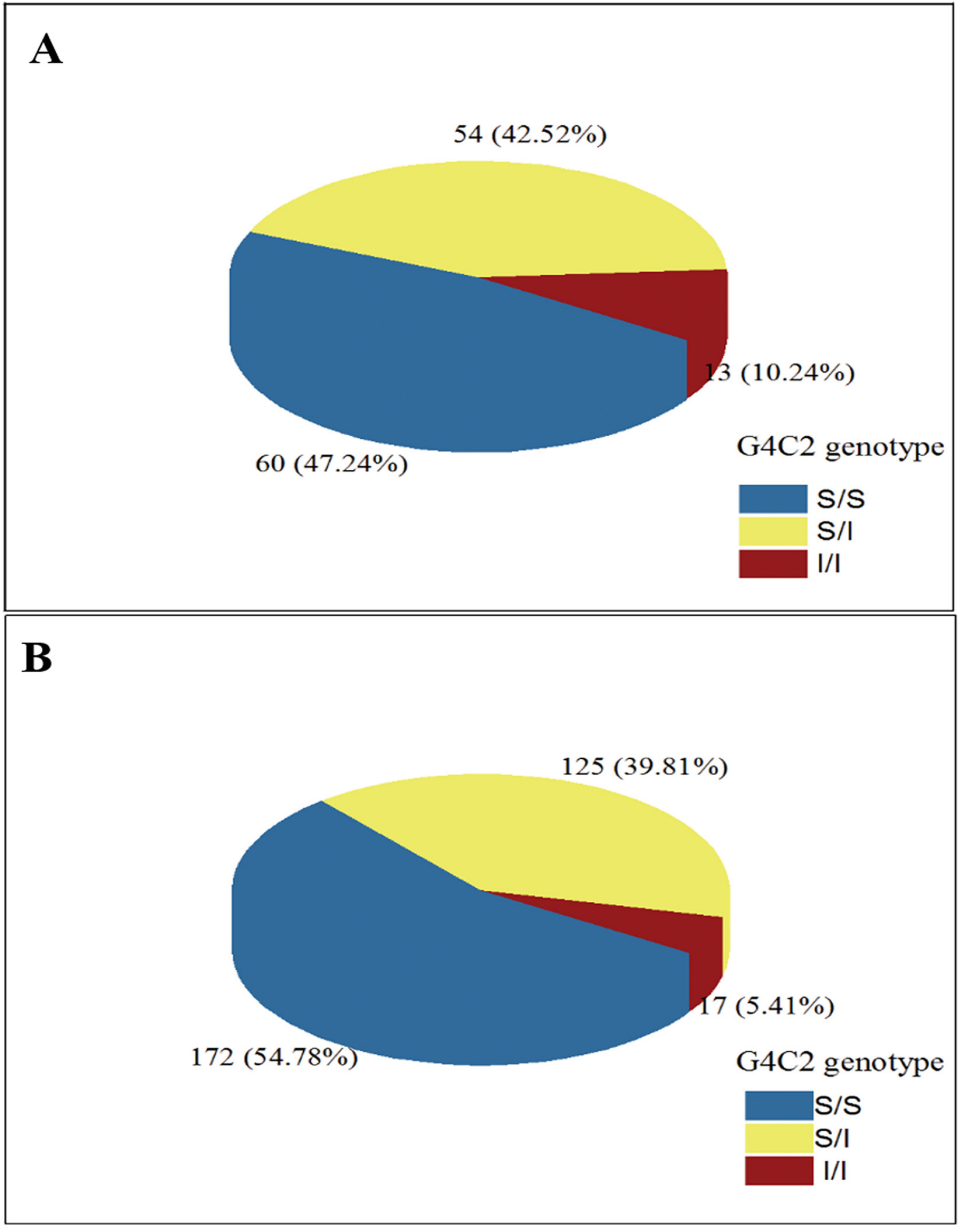
Frequencies of C9orf72 genotypes. A presents the frequencies in SCA3/MJD patients. B presents the frequencies in controls.

Table 1 presents the demographic information for this study. The accurate numbers of CAG repeats in the SCA3/MJD patients ranged from 64 to 84, as determined by the direct sequencing of recombinant DNA. No pathological repeat expansions of the *C9orf72* gene was detected in any of the individuals. The range of the repeat expansions in the SCA3/MJD patients was 2–18 units, and the most frequent number of repeats in all of the subjects was 2, followed by 6, 7, and 8 (S1 File). There was a difference when compared with that of Europe [21], where the most frequent repeats in Europe was 2 units, followed by 5 and 8. No significant difference was found in the distribution of repeats numbers in an individual’s larger allele between SCA3/MJD patients and normal controls (Fig 1 and Fig 2; more details in S1 File). We found that 142 of the 314 healthy controls (45.22%) harbored the intermediate sized *C9orf72* allele (range, 7–29), whereas 67 of the 127 patients with SCA3/MJD (52.76%) also harbored the intermediate-sized allele; this difference is not statistically significant.

**Table 1:** Demographic information and average number of repeats between SCA3/MJD patients and controls.

Variables	SCA3/MJD patients	Controls	p value
Case, n	127	314	-
Male, n (%)	72 (56.7)	173 (55.1)	0.83 ^a^
Age, Mean ± SD (years)	37 ± 9	36 ± 9	0.08 ^b^
Number of G4C2 repeats, Mean ± SD (size)	6 ± 3	5 ± 3	0.47 ^c^
Intermediate allele, n (%)	67 (52.76)	142 (45.22)	0.17 ^a^

^a^ x ^2^ test.

^b^ t-tests.

^c^ Mann-Whitney U test.

**Fig 3:**
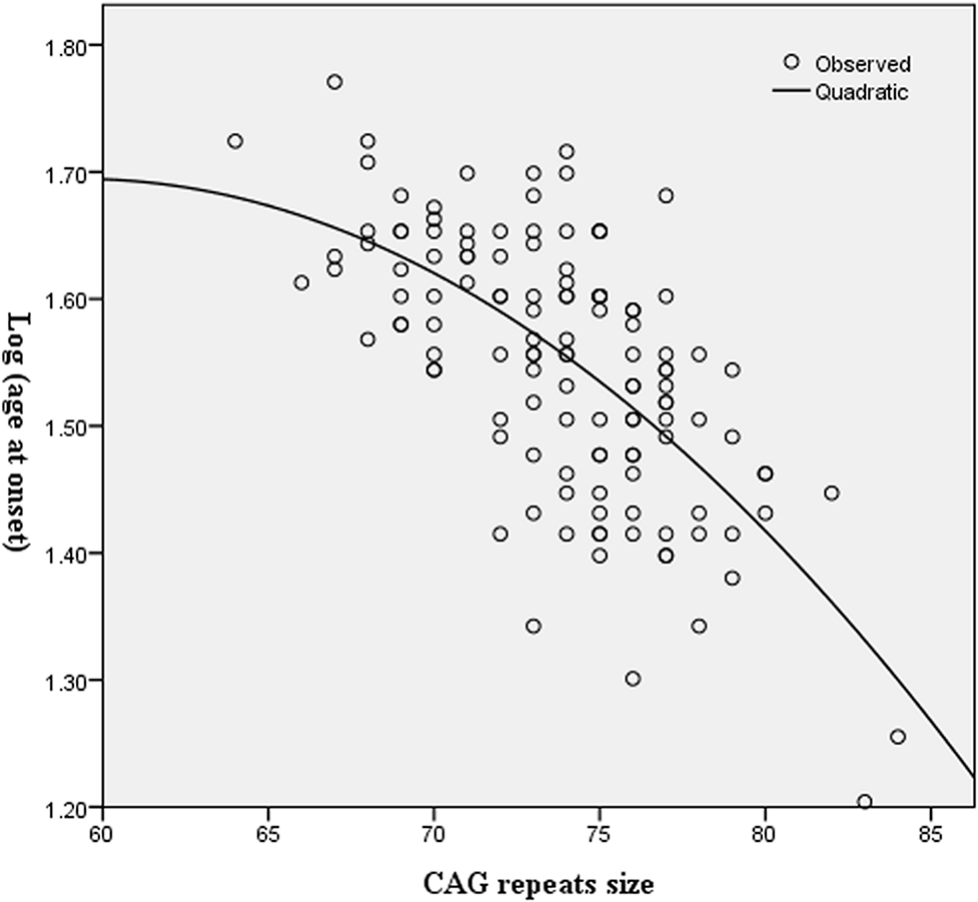
Modification of the age at onset due to expanded CAG repeats in *ATXN3* of SCA3/MJD patients. The X-axis denotes the expanded CAG repeat lengths and the Y-axis indicates logarithmically transformed ages at onset. Model parameters: Log (age at onset) = -0.571 + 0.076 Exp—0.001 Exp^2^. With Exp = Expanded CAG repeats in *ATXN3*.

The log age at onset was determined by the size of the expanded allele in SCA3/MJD patients, the determinant coefficient was 0.435. Significant quadratic effects of the *ATXN3* expanded alleles were found (negative effect P<0.0001, Fig 3). When larger repeat allele was used as a continuous variable and the expanded CAG repeats size in the *ATXN3* allele as a control variable, the partial correlation analysis showed that the size of the GGGGCC repeats was not correlated with the adjusted age at onset. No significant interaction between GGGGCC repeats and expanded CAG repeats was observed before performing covariance analysis. Interestingly, we observed patients with the intermediate sized allele of the GGGGCC repeats presented a 3-year earlier onset compared with that obtained with the short-sized allele group (Table 2). In multivariate linear regression analyses, when *C9orf72* genotype was taken into consideration the percentage of explanation of the onset variance increased from 45.7% to 46.4%. However, when gender was included as a variable, the model was not significantly improved.

**Table 2:** Descriptive statistics of the age at onset analysis of SCA3/MJD patients.

Variables	*C9orf72* genotypes		*p* value
	intermediate allele *	short allele *	
Patients, n (%)	67 (52.76)	60 (47.24)	-
Male, n (%)	36 (28.35)	36 (28.35)	0.59 ^a^
Age at onset, Mean± SD (years)	36 ± 8	35 ± 8	0.46 ^b^
Adjusted age at onset**, Mean ± SD (years)	34 ±1	37 ± 1	0.00 ^b^
Expanded CAG repeat length, Mean ± SD, (size)	74 ± 3	75 ± 3	0.06 ^b^

^a^ x ^2^ test.

^b^ t test.

^*^ The SCA3/MJD patients were classified into two genotypes according to the longer allele of the GGGGCC repeat: intermediate alleles (the repeat size ≥ 7 units) and short alleles (the repeat size < 7 units).

^**^ Adjusted for the size of the expanded CAG repeats in SCA3/MJD patients.

## Discussion

The GGGGCC repeats in intron 1 of the *C9orf72* gene were recently identified not only as a major cause of ALS and FTLD but also as a modifier in the pathogenesis of PD and AD [19, 20, 22, 23]. Previous studies indicated that neurodegenerative disorders exhibit clinical phenotype overlap and share common molecular mechanisms. In this study, we investigated the GGGGCC repeats mutation of the *C9orf72* gene and its association with SCA3/MJD patients. No large expansion was identified in our cohort, which suggests that large GGGGCC repeats of the *C9orf72* gene do not play a causative role in the pathogenesis of SCA3/MJD. Preliminary evidence suggested that the *C9orf72* mutation rates in patients with clinically diagnosed ALS/FTD in China, Japan, Korea, and Taiwan were much lower than that observed in Caucasian populations [16]. Here, we found a difference of the intermediate allele distribution between China and Europe. It is implied that the number of repeats varied greatly due to different nationalities and ethnicities.

The so-called polyglutamine diseases share the feature with each other: a negative relation between age at onset and the number of repeats in the expansion. However, the repeats length only explains 50–80% of the variability of age at onset, suggesting that other genetic factors contribute to the variability [24]. As the variability in age at onset is not completely explained by the effects of the causative and modifier sister genes, other genetic or environmental factors must also play a role. Great efforts have been devoted to the study of genetic links between neurodegenerative disorders. Previous study [2] reported biological interactions between some of the proteins involved in ataxias. The CAG tract of SCA2 gene interferes with MJD phenotype [25]. Recently, after a study of a large cohort of different SCAs, the authors reported that the polyglutamine genes interact with each other in SCA diseases to modify age at onset even when they contain a number of repeats considered to be normal [26]. In our study, we employed two analytical approaches for association testing, the larger repeat allele as a continuous variable and two genotyping categorical variables based on the individual’s short or intermediate alleles. However, only when the *C9orf72* gene as categorical variables can a significant result be found. There might be two reasons for the difference. Firstly, the sample of SCA3/MJD is not large enough to find the positive results. Secondly, the intermediate alleles act as a contributor to decrease the age at onset of SCA3/MJD patients. Then, how the intermediate alleles act as predisposing alleles and what is the pathogenic pathway of them in SCA3/MJD patients should be addressed in further studies. We found that the presence of the intermediate allele of the GGGGCC repeats in the SCA3/MJD patients decreased the age at onset by nearly 3 years. These results show that the length of the GGGGCC repeats in the *C9orf72* gene influence the SCA3/MJD phenotypes, i.e., larger repeats may cause earlier onset, which indicates that the expansion of the GGGGCC repeats exerts a modifying effect on SCA3/MJD disease diversity. That is to say, our results likely suggest a potential adjustment function between different genome structures to influence the disease. The data from this study support the hypothesis that the pathogenesis of diverse neurodegenerative disorders arises via interactive mechanisms, which may yield common therapeutic targets. This interaction thus provides new information that may aid both the understanding of the common pathogenic mechanisms associated with this class of neurodegenerative disorders and the identification of candidate modulators for these diseases.

In conclusion, our study provides the first demonstration that the larger GGGGCC repeats are associated with an earlier age at onset of SCA3/MJD. These data indicate that the development of SCA3/MJD may involve some physiological functions of the *C9orf72* gene and support the hypothesis that a specific mutation identified in one of the neurodegenerative disorders may be a modulator in this disease class [3, 20, 27]. Further studies, particularly on the interaction between *C9orf72* and ataxin-3, are needed to verify our results.

## Supporting Information

S1 File Raw data of longer allele of the GGGGCC repeats in SCA3/MJD patients and controls. (XLS)
